# Lenvatinib-Associated Cervical Artery Dissections in a Patient with Radioiodine-Refractory Metastatic Papillary Thyroid Carcinoma

**DOI:** 10.3389/fmed.2017.00220

**Published:** 2018-02-23

**Authors:** Phillip J. Groden, Thomas C. Lee, Shamik Bhattacharyya, Jean Connors, Jochen Lorch

**Affiliations:** ^1^Medical Oncology, Dana-Farber Cancer Institute, Boston, MA, United States; ^2^Department of Radiology, Brigham and Women’s Hospital, Boston, MA, United States; ^3^Department of Neurology, Brigham and Women’s Hospital, Boston, MA, United States; ^4^Hematology Division, Brigham and Women’s Hospital, Boston, MA, United States

**Keywords:** cardiovascular, thyroid, cancer, arterial dissections, tyrosine-kinase inhibitors

## Abstract

Lenvatinib is a tyrosine kinase inhibitor (TKI) approved by the FDA for the treatment of radioiodine-refractory (RAIR) thyroid cancers. Side effects can be severe, however, and include headaches, hypertension, arterial and venous thromboembolic events, and fatalities. Cervical artery dissections (CADs) are leading contributors of cerebral ischemia in young adults, yet the pathophysiology is poorly understood. Here, we describe a case of a 34-year-old female with recurrent, metastatic, RAIR papillary thyroid cancer who, following her second week of lenvatinib treatment, developed significant CAD which resolved following the termination of the TKI therapy. Given the lack of risk factors for the disorder in the patient’s history, the known cardiovascular events associated with the drug, previously described cases of arterial dissections linked to VEGF inhibitors, and the temporal relationship between the onset of symptoms and the treatment start date, a causal relationship between the CAD and lenvatinib is suggested.

## Introduction

Differentiated thyroid carcinomas—which include papillary (PTC) and follicular (FTC) thyroid cancer as well as poorly differentiated variants—make up roughly 90% of all thyroid cancers. While most cases are cured with a combination of surgery and radioiodine (RAI) therapy, a subset of patients will develop radioiodine-refractory (RAIR) incurable disease (1). Prognosis remains poor for these patients with a 10-year survival rate of just 10% (2).

Lenvatinib is a tyrosine kinase inhibitor (TKI) that targets numerous signaling pathways linked to the growth and invasion of thyroid cancer, including vascular endothelial growth factor receptors (VEGFRs) 1, 2, and 3; fibroblast growth factor receptors (FGFRs) 1, 2, 3, and 4; platelet-derived growth factor receptor (PDGFR) α; and both RET and KIT proteins. The drug was approved by the FDA for the treatment of RAIR thyroid cancers based on data from a phase 3, double-blind study in 392 RAIR patients with differentiated thyroid carcinomas. The results of the trial demonstrated a significant difference in progression-free survival (PFS) in the treatment arm compared with placebo subjects (18.3- vs. 3.6-month median PFS, respectively) (2).

Adverse events experienced during the trial were significant. Among participants on lenvatinib, 97.3% experienced treatment-related adverse events, with 75.9% classified as Grade 3 or higher. Vascular events identified as Grade 3 or higher within the treatment group included hypertension (41.8%), arterial thromboembolic events (2.7%), and venous thromboembolic events (3.8%); other adverse symptoms, including headaches (27.6%), were also commonly reported. Notably, 2.3% of lenvatinib treated patients developed adverse events during the course of the study that proved fatal and were considered by investigators to be treatment-related, including an instance of both pulmonary embolism and hemorrhagic stroke (2).

Cervical artery dissections (CAD) are rare, affecting less than 1% of individuals in North America per year (3). Since cases that do not present with clinical symptoms prior to ischemic events may go undiagnosed, experts believe that the estimated incidence of CAD is actually underreported (4). However, the disorder remains a leading contributor of cerebral ischemia within young adults. Dissections may occur in either the vertebral or—most commonly—internal carotid (ICA) arteries and are characterized by mural arterial wall hematomas caused by the diversion of blood-flow through a breach in the intimal membrane (5). Strokes can occur either from arterial occlusion or from embolism *via* thrombus formation at the site of dissection. Late complications include pseudoaneurysm formation and potentially arterial rupture.

At present, the pathophysiology of CAD is not well understood. It has been suggested that patients who develop CAD are predisposed to the disorder due to a genetically determined defect within their vessel walls (6), which could explain CAD patients’ frequent simultaneous presentation with multiple arterial abnormalities (4). Underlying inherited connective tissue (CT) disorders may predispose patients to CAD events. Approximately half of all CAD patients exhibit dermal irregularities of CT (6), and cases have been associated with certain monogenic CT disorders, such as Marfan’s syndrome, osteogenesis imperfecta, and Ehlers–Danlos syndrome (7, 8). Furthermore, multiple studies have discovered associations between CAD and specific genetic mutations, including ICAM1, COL3A1, and MTFH (which encode intercellular adhesion molecule 1, collagen type-III α-1, and 5–10 methylenetetrahyrdrofolate, respectively) (9–13).

Major traumatic events are also significant contributors to CAD; up to 2% of all cases of blunt trauma will present with dissections (14). Non-traumatic (i.e., spontaneous) CADs have been linked to a number of other conditions, including hyperhomocysteinemia (9, 10, 15–17); decreased concentration of α-1 antitrypsin (18); recent acute infection (19); arterial hypertension (11, 20); frequent migraines (21, 22); and use of oral contraceptives (23). Unknown genetic factors could play a role in predisposing individuals to dissections, as CAD can affect members of the same family lacking a history of heritable CT disorders (24). Previous reports of arterial dissections on anti-VEGF therapy include two cases of aortic dissection on sunitinib and axitinib, respectively (25, 26). There exists only one reported case of CAD with a suspected connection to anti-VEGF therapy: a 60-year-old male who received sunititnib for metastatic renal cell cancer (27). Here, we describe a case of multiple CAD in a patient who received lenvatinib for RAIR PTC.

## Patient

The patient—who has provided consent for the publication of this report—is a 34-year-old female with a history of hypothyroidism that was diagnosed with localized PTC in November of 2013. In January of 2014, the patient underwent a total thyroidectomy and modified bilateral neck dissection, revealing an 8.4 cm × 4.5 cm × 2.5 cm mass that had replaced the entire right thyroid lobe, the isthmus, and a majority of the left lobe; additional pathologic findings in the background of chronic lymphocytic thyroiditis were consistent with the diagnosis of sclerosing variant PTC. Surgical margins were positive, and 35 out of 63 extracted lymph nodes contained PTC. Following surgery, the patient received 158 mCi of iodine-131 (RAI therapy) in March of 2014, and posttreatment scans demonstrated uptake in the thyroid bed without evidence of distant metastatic disease.

In May of 2015, the patient underwent a modified bilateral neck dissection, and conglomerates of lymph nodes were excised from level VI bilaterally and from left level IIB. Thirty-nine of 42 right cervical lymph nodes were positive for recurrent PTC. Following surgery, the patient received 150 mCi of iodine-131 in July of 2015. Though increased uptake consistent with nodal metastases was noted in the mid-neck, the posttreatment scan again showed no RAI avid distant metastatic disease.

Her disease recurred in January of 2016 when a CT scan of the patient’s neck noted numerous enhancing nodes that had increased in size from prior examinations, including an abnormal, heterogeneous left level IIB node (1.4 cm × 0.8 cm), two left-level IV nodes, and several upper mediastinal nodes. Because the patient was reluctant to undergo further surgical intervention, she was prescribed lenvatinib at 14 mg daily in March of 2016 with plans to escalate her dose as tolerated.

**Figure 1:**
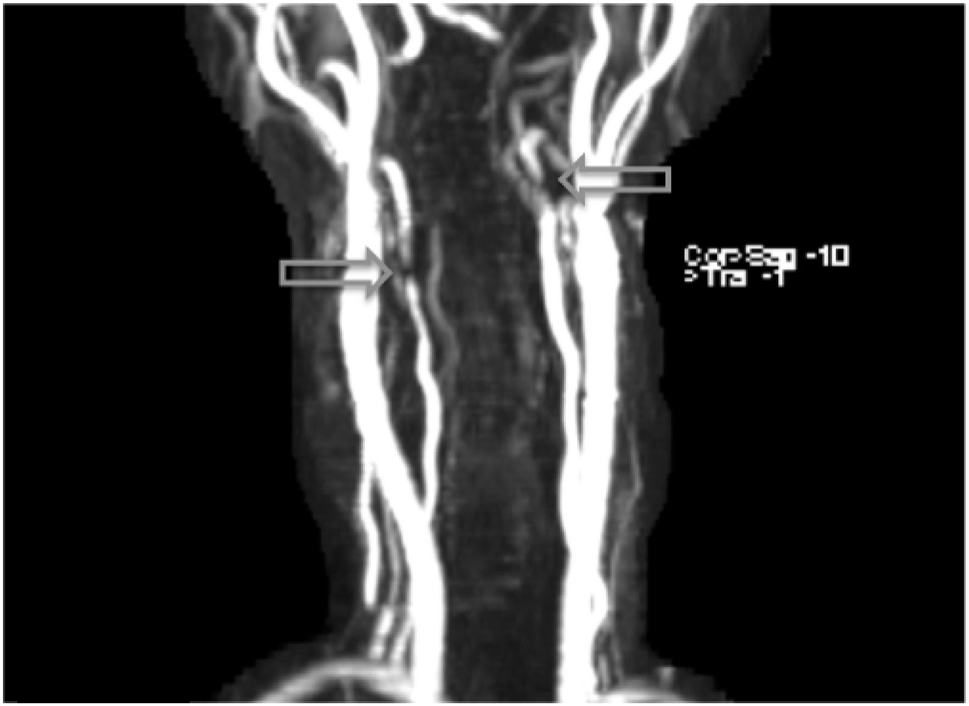
Arterial dissections noted in both vertebral arteries on MRI angiography following the patient’s second week on lenvatinib (14 mg) treatment.

**Figure 2:**
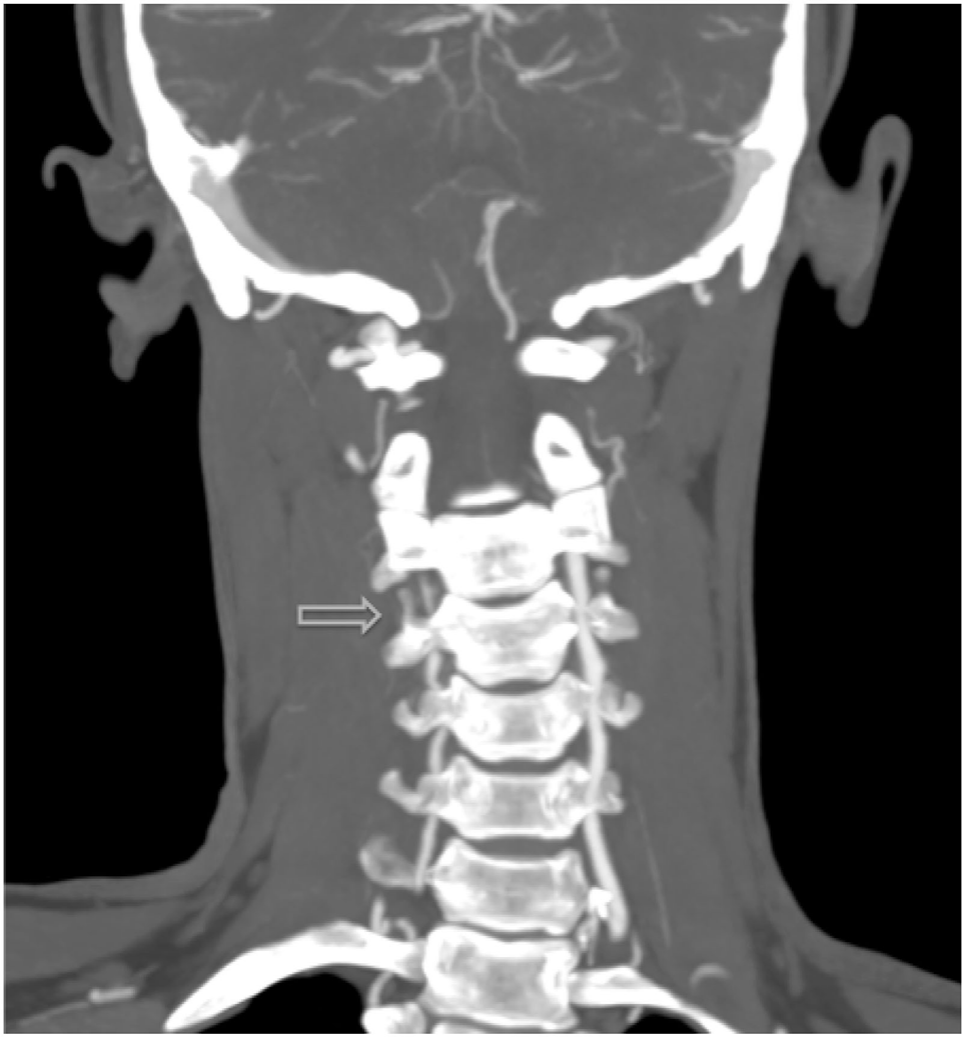
Additional MRI angiography imaging revealing bilateral focal loss of flow-related enhancement in the distal cervical vertebral arteries with distal antegrade flow compatible with bilateral vertebral dissections with severe stenosis and antegrade collaterals.

**Figure 3:**
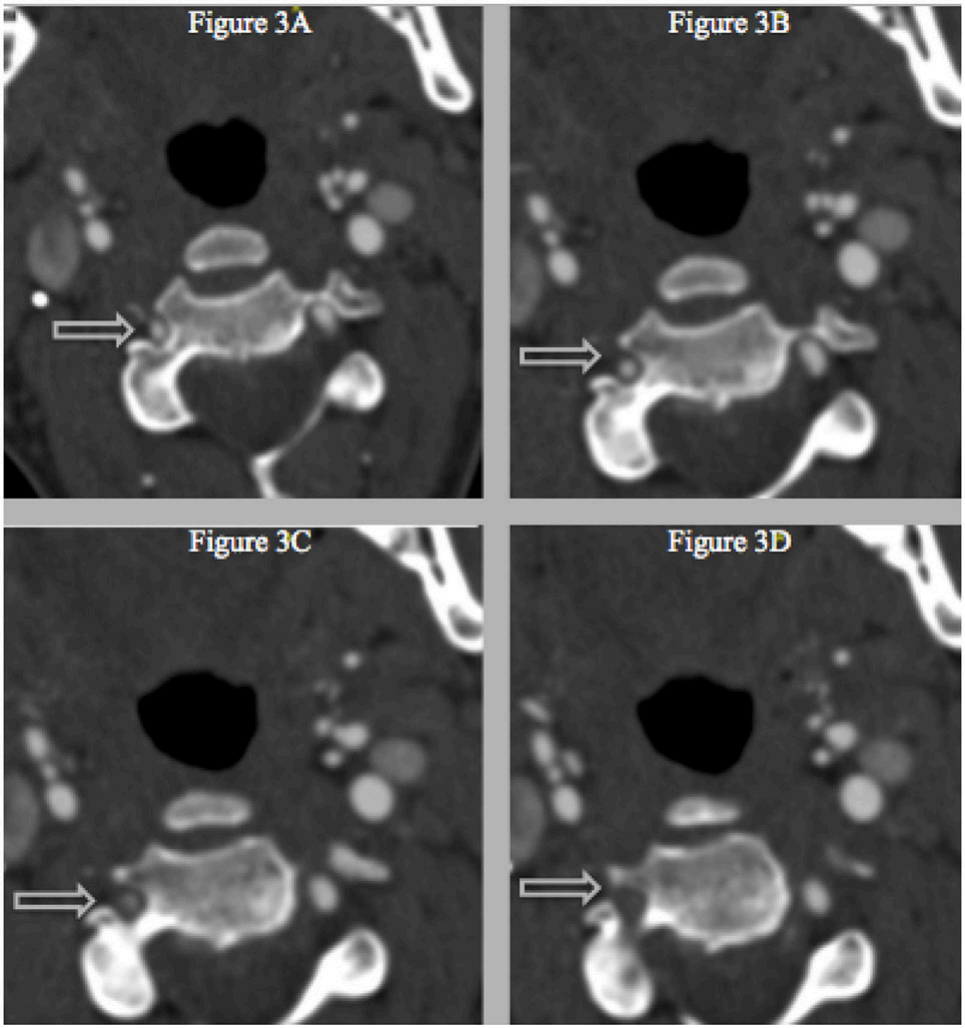
(A–D) Successive CT with IV-contrast images (0.75 mm) depicting a segment of non-opacification within the right vertebral artery and a normal vertebral canal at level C3, which suggests that the stenosis is not related to vascular hypoplasia within that area.

**Figure 4:**
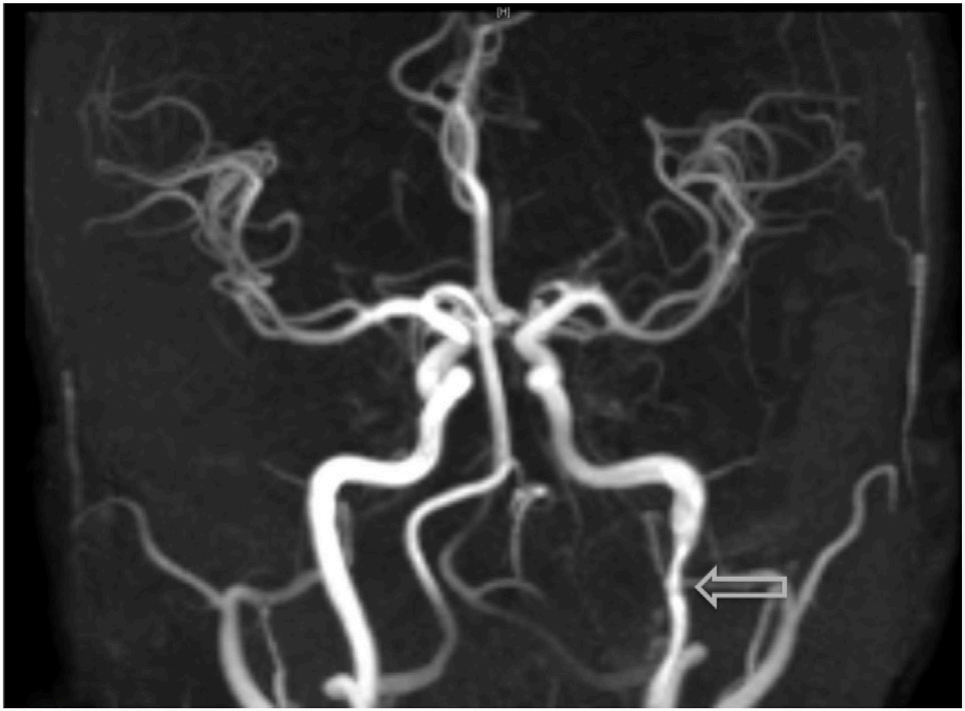
Visualization of an additional dissection noted within the left-internal carotid artery on MRI angiography, an examination which was prompted due to the onset of acute headaches.

**Figure 5:**
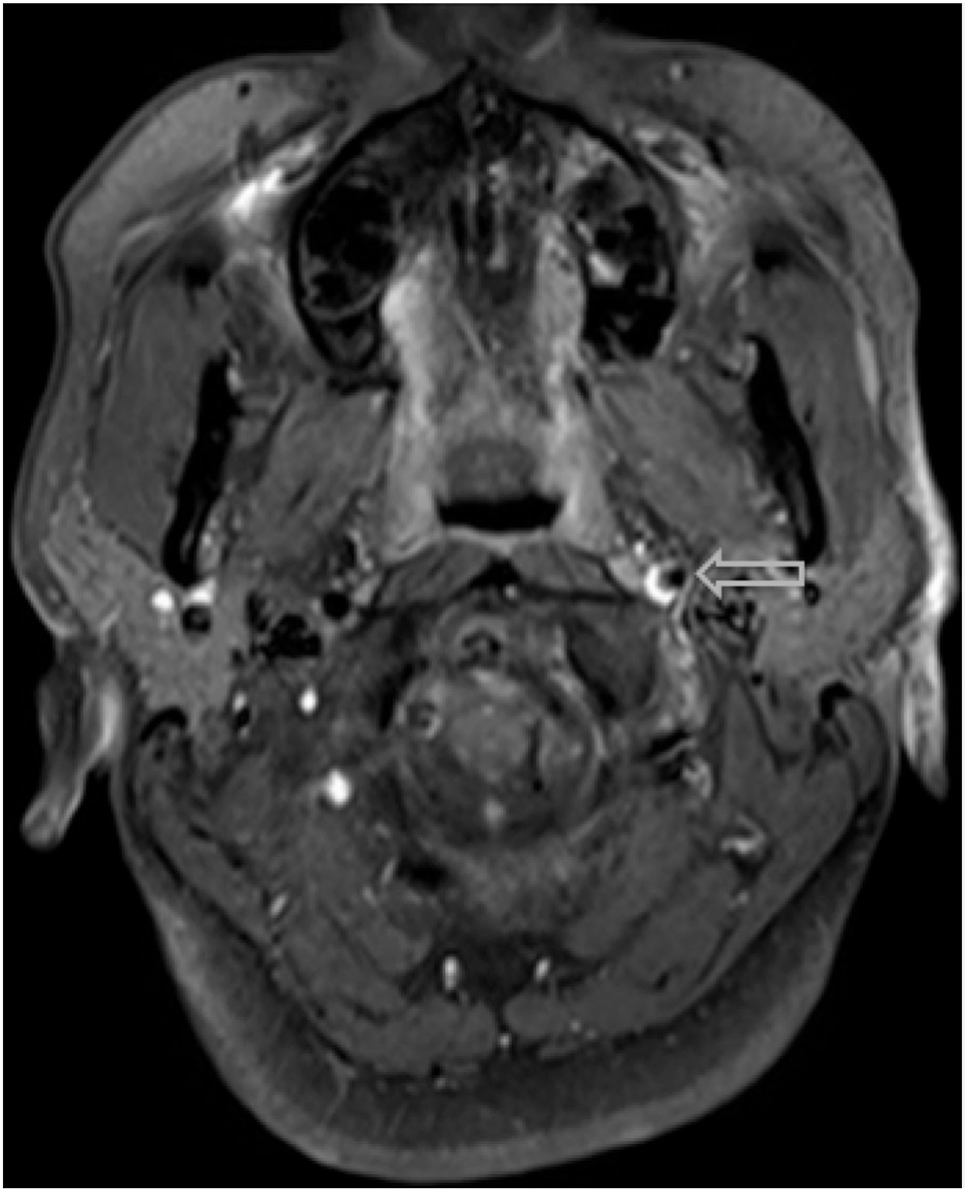
A hyper-intense, crescent-shaped signal noting the presence of an acute thrombus within the cranial arteries on MRI angiography.

**Figure 6:**
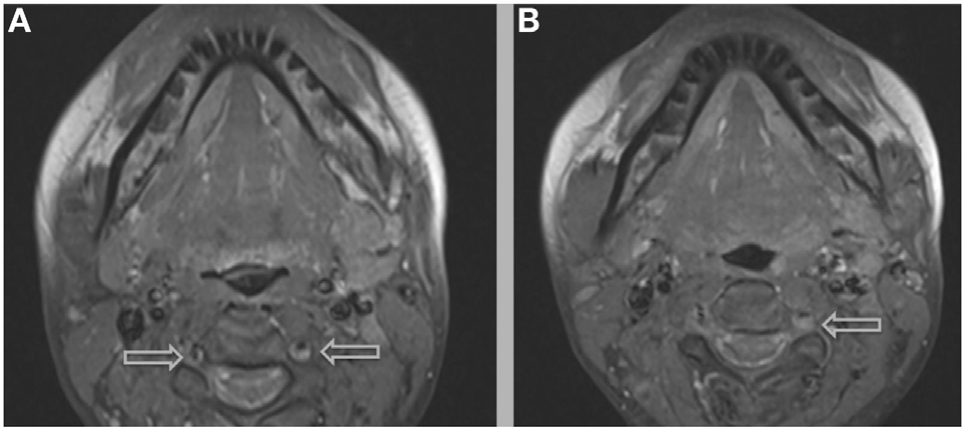
Additional MRI imaging indicating mural hematomas within the right **(A,B)** and left **(B)** vertebral arteries, findings that would resolve following anticoagulant therapy and the cessation of lenvatinib.

Within 3 weeks of starting the drug (treatment day 16), the patient began to experience severe acute onset neck pain and headaches, prompting her to contact her oncologic team for assistance. Concerned with these symptoms, both a fat-saturated T1-weighted MRI of the brain and an MRI angiogram (MRA) of the cervical and cranial vessels were performed, revealing dissecting aneurysms in both the vertebral (Figures 1–3) and left-internal carotid (Figure 4) arteries in addition to T1 crescent-shaped hyperintense methemoglobin in the dissected walls, indicating mural hematomas (Figures 5 and 6). In response to these findings, the lenvatinib treatment was discontinued and the patient was placed on indefinite anticoagulation therapy.

**Figure 7:**
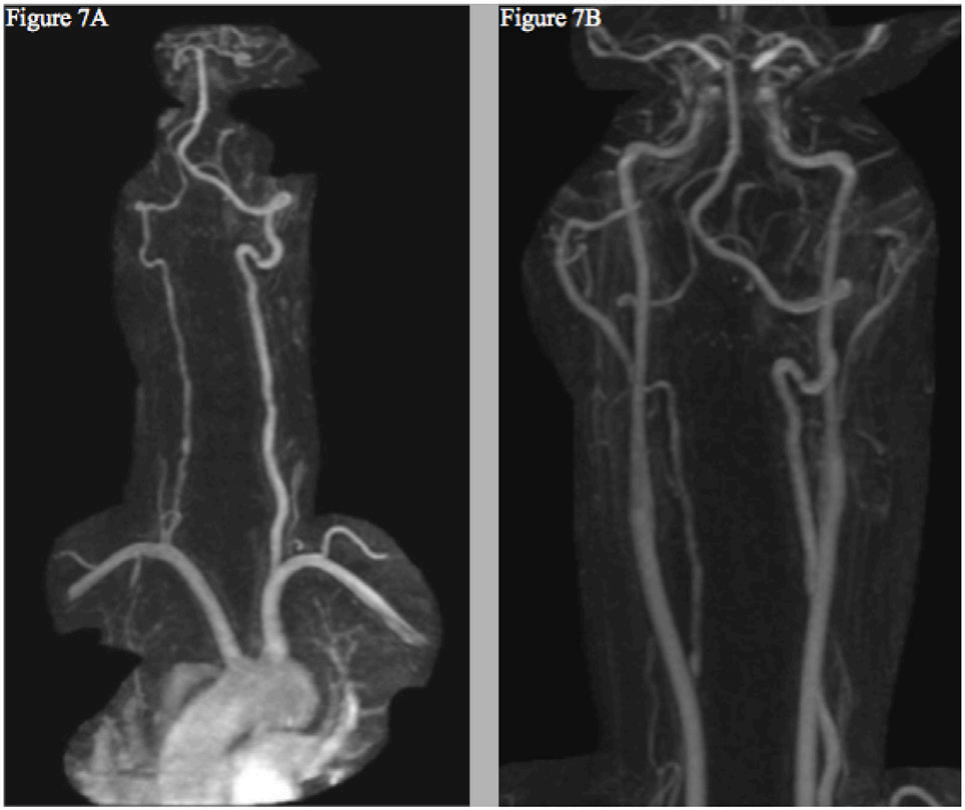
Follow-up MRI angiogram showing interval improvement of the left vertebral **(A)** and internal carotid arteries **(B)**—without evidence of new dissection or aneurysm—and persistent right cervical vertebral artery contour irregularity **(A)**.

To date, the patient’s symptoms have resolved without neurologic deficits. Repeat MRI and MRA scans performed in July of 2016 displayed improvement of both the left ICA and vertebral artery, though the right vertebral artery remained diffusely narrower than the prior scan (Figure 7). Given the known association with CAD, sequencing of COL3A1, ICAM1, and MTHFR genes was performed [Fulgent Diagnostics™ (Temple City, CA, USA) https://www.fulgentgenetics.com], yet no mutations were revealed.

This is the first report of CAD in a patient on lenvatinib. While a causal link between lenvatinib and CAD cannot be established, the temporal relationship between the onset of the patient’s symptoms and the acute radiographic findings suggests that the drug was a contributor to this process.

## Discussion

The most common symptom presented by patients suffering from CAD is severe head and/or neck pain and ischemic events, such as a transient ischemic attack (TIA) or cerebral infarct. However, since patients may suffer from a CAD-related ischemic insult without first experiencing any clinical symptoms, it is believed that the incidence of CAD, reported as less than 1%, is appreciably underestimated. Other signs of CAD include Horner’s syndrome, cranial nerve palsy (both resulting from ICA dissections), or cervical-root injury (vertebral artery dissections) (4). Visualized on a radiologic exam—most commonly *via* MR or CT-angiography—CAD may present in differing manners, such as a dissecting aneurysm (17%) (Figures 1–4), a tapered occlusion (35%), or a tapered stenosis (48%) (3). Mural hematoma, the chief radiographic finding of CAD, can be identified by a hyperintense, crescent-shaped signal encompassing a diminished lumen within an expanded artery (Figures 5 and 6). Other noticeable signs of CAD on radiologic examination include the following: long tapered stenoses, false lumens, and luminal flaps (4).

For patients suffering from CADs, early treatment of the disorder with antiplatelets or anticoagulants is considered critical to prevent ischemia and recurrence, although data are sparse (4). Within the first year, roughly 82% of individuals will have either a complete resolution or display stable residual radiographic irregularities; (3) patients who have not suffered an ischemic event have the highest likelihood of total recanalization (28). Among patients who present with ischemic stroke or arterial occlusion following a dissection, mortality rates of 20% and higher have been reported (29). Though recurrent ischemic events can occur—most commonly within the first month after diagnosis—such episodes are generally rare (4, 30, 31), and recurrent dissections following the initial insult are also unlikely (32). In the case presented here, no recurrence has occurred and the patient is past one year since the onset of her symptoms.

Lenvatinib has been associated with numerous serious vascular complications, including severe hypertension, thromboembolic events, and hemorrhagic strokes (2). Review of the published literature revealed three cases of arterial dissections that occurred while on TKI’s similar to lenvatinib: axitinib (one report) and sunitinib (two reports), both of which are strong inhibitors of VEGF. Of these cases, two patients—treated for metastatic renal cell and gastrointestinal stromal cancer with axitinib and sunitinib, respectively—were diagnosed with aortic dissections, one of which being a dissection of the coronary artery (25, 26). The sole report of CAD while on anti-VEGF-TKI treatment describes a 60-year-old male with metastatic renal cell cancer who had been on sunitinib for several months; notably, the patient was found to have hyperhomocysteinemia, a known risk factor for CAD (27). Our case differs in that the patient is young, received anti-VEGF treatment for only a short period of time, and had none of the known risk factors for arterial wall instability, including genetic markers COL3A1, ICAM1, or MTHFR.

As previously mentioned, major traumatic events are known contributors to CAD; in addition, multiple CAD—as seen with our patient—is more frequently associated with previous head and neck surgery in comparison to individuals with a single dissection (33). Though the patient had undergone surgeries requiring hyperextension of the neck, her last procedure had been over 10 months prior to her CAD diagnosis, and she had not experienced any unusual symptoms in the aftermath of these surgeries. In addition, a restaging MRI was completed roughly one month before starting lenvatinib treatment, and no intramural thrombus or obvious narrowing of the arteries was noted. While an occult injury related to previous neck surgeries cannot be ruled out as a potential contributor to her CAD, the course of her treatment was typical of those lenvatinib is approved for: patients with RAI-refractory thyroid cancer. Because of this, providers who care for similar patients should be aware of the potential association between the TKI inhibitor and CAD within this population.

Though a causal relationship between lenvatinib and CAD is not firmly established, the previous reports of arterial dissections linked to similar TKI’s, the absence of risk factors for the disorder in the patient’s history, the temporal relationship between the patient’s symptoms and the treatment start, and the resolution of these symptoms and improvements of radiographic findings following discontinuation of the drug (Figure 7) all suggest that the drug may have played a direct role in the development of the multiple CAD.

To the best of our knowledge, this is the first report of CAD associated with the FDA-approved lenvatinib. In symptomatic patients, similar steps should be taken immediately to address this potentially life-threatening complication.

## Ethics Statement

Written informed consent was obtained from the patient for the publication of this case report and any accompanying images.

## Author Contributions

PG is the primary author and conducted the necessary background research for the writing of this paper. TL assisted in providing the imaging and his interpretation of these findings for crucial figures included within the manuscript. SB and JC gave their expert opinions on the topics discussed in the paper, including interpretation of the relevant imaging and background on CADs, respectively. JL is the patient’s treating physician and provided guidance throughout the creation of this manuscript. All authors read, edited, and approved the final manuscript prior to submission.

## Conflict of Interest Statement

The authors declare that the research was conducted in the absence of any commercial or financial relationship that could be construed as a potential conflict of interest. The reviewer JH and handling editor declared their shared affiliation.
